# Is 48 h a critical cut-off point for mortality in geriatric hip fractures?

**DOI:** 10.3906/sag-2003-194

**Published:** 2020-10-22

**Authors:** Ahmet Emre PAKSOY, Kerim ÖNER, Ferdi POLAT, Serhat DURUSOY

**Affiliations:** 1 Department of Orthopaedics and Traumatology, Faculty of Medicine, Ataturk University, Erzurum Turkey; 2 Department of Orthopaedics and Traumatology, Faculty of Medicine, Bozok University Yozgat Turkey; 3 Department of Anesthesiology and Reanimation, Yozgat State Hospital, Yozgat Turkey

**Keywords:** Hip fractures, time factors, response time, orthopedic surgery, mortality

## Abstract

**Background/aim:**

In this study, our objective was to evaluate the mortality in geriatric hip fracture patients who were operated within 48 h after admission or after the 48th h.

**Materials and methods:**

A total of 194 patients who had undergone surgery for hip fracture between 2016 and 2018 were retrospectively evaluated. Patient information was obtained from the hospital’s database using the ICD codes 81.52, 82.00–82.09, and 82.10. Radiological examination reports were collected from the patient files. Information on mortality was obtained from the Death Notification System of the Turkish Ministry of Health. First-year mortality rates of patients operated within 48 h (Group 1) and those operated at 48–96 h (Group 2) were compared.

**Results:**

The mean duration between admission to the hospital and surgical intervention was 33.90 ± 1.95 h (3–96 h). The mean total hospitalization time was 7.29 ± 1.53 days (2–36 days). Of the patients, 62 (32%) died within one year after the operation. The mean survival times for patients operated ≤48 h or >48 h were 8.47 ± 1.90 and 6.57 ± 2.59 months, respectively (Z = 1.074, P = 0.283). There was no significant correlation between survival time and the time delay before the operation (r = –0.103, P = 0.153). Additionally, the Cox regression analysis, including age (years), ASA (grade 3 vs. 2), time to operation (h), and days spent in the ICU, demonstrated no significant independent effect of the time to operation on survival (P = 0.200).

**Conclusion:**

Although shortening the time to surgery may have some rationale, we did not find any difference in patients operated before 48 h compared to 48–96 h concerning mortality.

## 1. Introduction

The world population is aging at a fast pace. Consequently, the annual number of hip fractures increases rapidly. Approximately 6.3 million cases of hip fractures are anticipated in 2050 [1]. Hip fractures are among the leading causes of morbidity and mortality in the geriatric population. In Europe, osteoporotic fractures accounted for more disability adjusted life years (DALYs) lost than most cancers [2]. 

Additionally, geriatric hip fractures are a significant source of the financial burden on health security systems. Also, there are other incalculable costs, such as the necessity of hospital attendance after surgery. Different clinical approaches are investigated for a solution to this health problem and its relationship to the increased rates of morbidity and mortality, as well as the high financial losses [3,4]. Different surgical and anesthetic methods and postoperative care approaches, used in varying combinations, are utilized in these patients. 

Although there are different opinions regarding the timing of the surgery for geriatric hip fractures, there is consensus that the time before surgery should be short [5–8] . However, opinions about on the critical cut-off point of the time delay before surgical intervention are varied [9]. American Academy of Orthopaedic Surgeons guideline recommends surgery within 48 h in geriatric hip fractures [10]. In this study, we focused on geriatric patients with hip fractures who were treated according to a model that includes early partial hip prosthesis surgery and routine postoperative intensive care unit (ICU) monitoring. Our objective was to evaluate the mortality in geriatric hip fracture patients operated before or after 48 h. 

## 2. Materials and methods

A descriptive cross-sectional study based on patient records was conducted. The research protocol was approved by the local ethics committee of the study hospital (IRB number: 2017-KAEK-189_2018.11.14_04). 

This retrospective study was conducted in a health center responsible for a settlement with a population of approximately 150,000, where no training hospital is available. It is the only hospital with an operating room in the central settlement area. Although it is not a referral center, the hospital welcomes yearly around 25,000 patients in the orthopedic clinics.

Using the hospital’s health registry, all hip fractures with the ICD-9 codes 81.52, 82.00–82.09, and 82.10 were screened retrospectively [11]. A total of 194 patients over the age of 65 years, who had undergone a cemented bipolar prosthesis due to unstable intertrochanteric fractures between 2016 and 2018, who were followed up for longer than one year, were included in the study. Patients with hip fractures under the age of 65 years, repeated fractures, revision surgeries, ICD code mismatch, and patients with osteosynthesis over the age of 65 years were excluded (Figure). Patient information was obtained from the hospital’s database, patient files, ICU notes, and radiological examination reports.

There was no other health center with an operating room and ICU near to the study hospital. Besides, none of the patients with ICD-codes 82.00–82.09, or 82.10 were referred to larger health centers during the study period. All the patients included were admitted first to the study hospital.

The patients were analyzed in two groups. Group 1 consisted of patients who were operated within 48 h after the incident, while Group 2 patients had undergone surgery between 48 and 96 h after the fracture. 

All patients were operated by the same two surgeons. The operation time was planned according to the medical condition of the patient. Surgery was arranged as early as possible, depending on the availability of the operation room. All patients had undergone spinal anesthesia and were monitored postoperatively by an anesthesiologist in the ICU room. The decision to discharge from the ICU was made by the anesthesiologist. Prophylactic treatment for deep vein thrombosis was initiated with low molecular weight heparin was started for all patients.

The mortality status of the patients was tracked via the Death Notification System of the Republic of Turkey Ministry of Health. This system contains information about whether any citizen is dead or alive. Death information is recorded on the same day. Depending on the information obtained from this system, patients were grouped as alive or dead.

**Figure:**
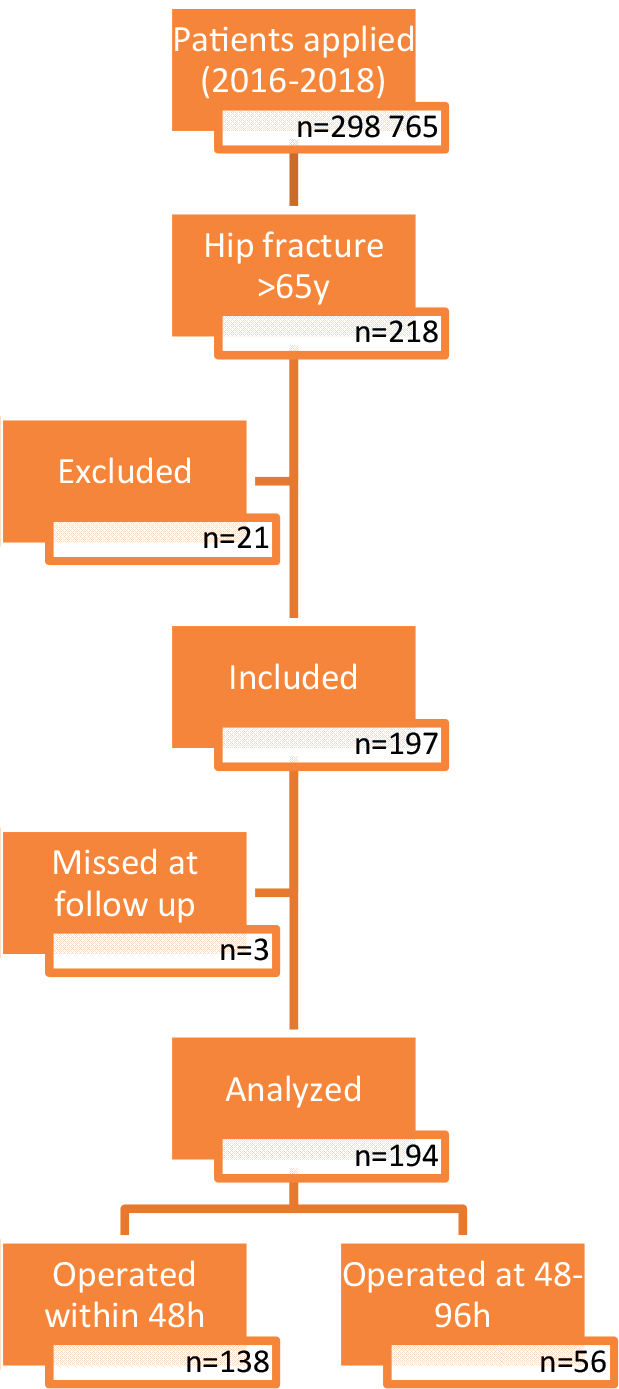
Study flow diagram.

### 2.1. Statistical methods

The statistical analysis was done with the Statistics Software Package (SPSS) version 22.0 (IBM Corp., Armonk, NY, USA). The normal distribution of numerical variables was checked with the Kolmogorov–Smirnov test. All variables except age were skewed. Logarithmic transformation was used to normalize the skewed data. The comparison between categorical variables was analyzed with the chi-square test. Pearson’s correlation test was used for checking the relationship of different numerical variables. The independent samples t-test (or Mann–Whitney U test) was used for the assessment of differences between groups. Cox regression analysis was applied to test for independent factors affecting survival time. A P-value < 0.05 was considered as statistically significant.

## 3. Results

The study sample consisted of 86 males (43.9%) and 108 females (55.1%). The mean age was 80.52 ± 6.96 years (61–95 years). Of the patients, 152 (78.4%) were admitted on weekdays and 42 (21.6%) on weekends. The American Society of Anesthesiologists (ASA) score was two in 92 (47.4%) patients and three in the remaining 102 (52.6%) patients. The mean duration between admission to the hospital and surgical intervention was 33.90 ± 1.95 h (3–96 h), while the median hospitalization in the ICU was 1.8 days (1–36 days). The postoperative complications were as follows: pulmonary infection (n = 11; 6%), superficial wound infection (n = 10; 5%), deep wound infection (n = 3; 2%), heart failure (n = 4; 2%), urinary tract infection (n = 12; 6%), and dislocation (n = 2; 1%).

The mean total hospitalization time was 7.29 ± 1.53 days (2–36 days). While 44 of the 138 patients (31.9%) in Group 1 died within the one-year follow up, 18 of the 56 patients (32.1%) died in Group 2 (chi-square = 0.001, P = 0.972). The survival time significantly decreased with higher ASA scores and older age (Table 1).

The relative delay of the operation in Group 2 was related to the availability of the operation room in most cases. Survival time was negatively correlated with age (r = –0.238, P = 0.001) and the time spent in the ICU (r = –0.335, P < 0.001). There was no significant association between survival time and the time delay before the operation (r = –0.103, P = 0.153). Additionally, the regression analysis, including age (years), ASA (grade 3 vs. 2), time to surgery (h), and days spent in the ICU, demonstrated no significant independent effect of the time to operation on survival (Table 2).

**Table 1:** Comparison of mean survival times between different groups.

		Survival time (months)		t#/Z*	P value
		Mean	SD		
Sex	Male	8.85	1.89	1.752*	0.080
	Female	7.11	2.29		
Age	<80 years	8.96	1.97	2.328*	0.020
	≥80 years	7.11	2.21		
ASA grade	2	9.83	1.73	3.380*	0.001
	3	6.44	2.34		
Day of admission	Weekday	7.84	2.18	1.115#	0.909
	Weekend	7.96	1.92		
Time to operation	48 h or less	8.47	1.90	1.074*	0.283
	>48 h	6.57	2.59		

*Mann–Whitney U test. # Independent samples t-test

**Table 2:** Cox regression analysis computer output.

	B	SE	Wald	P	Exp(B)	95.0% CI for Exp(B)	
						Lower	Upper
Age (years)	0.045	0.021	4.331	0.037	1.046	1.003	1.090
ASA (grade 3 vs. 2)	0.730	0.297	6.056	0.014	2.075	1.160	3.710
Time to operation (h)	0.568	0.444	1.639	0.200	1.764	0.740	4.209
ICU stay (days)	0.956	0.466	4.210	0.040	2.601	1.044	6.484

## 4. Discussion

This study showed a significant relationship between survival time and the gap before the operation in the univariate analyses. However, multivariate analysis demonstrated no significant independent effect of time to significant independent on survival. Instead, age, ASA grade, and days spent in the ICU had substantial impacts on survival time.

Hip fractures are common and worldwide important health concerns among the elderly. Geriatric hip fractures cause significant financial losses, affect the patients’ social environments, are frequently fatal, and are a public health concern [2, 12,13] . Surgery is the preferred treatment option for the majority of these patients [3].

In-hospital and one-year mortality rates are 9% and 20%–34%, respectively [6, 14–16]. In our study, we found comparable rates (2% and 29%, respectively). On the other hand, a high proportion of comorbidities are expected to be present in males, and ASA score and time to surgery are given as the primary risk factors for increased mortality [6]. Among these factors, the only one in which surgeons can intervene is the time between admission and surgical intervention. In hip fracture patients, factors such as a high number of comorbidities, electrolyte imbalance, medication, and attempts to optimize the conditions are the primary reasons for delaying the surgery [17].

On the other hand, the early intervention significantly decreases the costs [12]. Problems such as the limited number and capacity of operating rooms lead to delayed surgery in most health centers. In patients who have to be referred to ICU after surgery, the reservation of a bed in ICU may also delay the surgical intervention.

It was suggested that a hip fracture should be considered as urgent as cardiac ischemia, recommending immediate surgery [15]. However, there are other studies in which it is advocated that early surgery does not have a definite impact on mortality [17]. The American Academy of Orthopedic Surgeons published its guidelines in 2015 in which it reported that only moderate evidence supports the statement “surgery < 48 h decreases mortality and morbidity in hip fractures” [10]. Besides, intervention within 24 h was found superior only regarding the hospital expenses [12]. In our study, although we did not find a significant difference in the survival times of the early intervened patients; this difference did not exist even after adjustment. Thus, we concluded that the interactions between age, ASA grade, and ICU stage have to be considered together before deciding on a significant effect of the intervention time on mortality. Likewise, Moran et al. [18] conducted a prospective study. They operated 99% of the patients with a hip fracture within the first 4 days and reported that delays of no longer than 4 days did not increase the mortality rate. Maheshwari et al. [15] grouped geriatric hip fractures according to the time until surgery as <18 h, 18–24 h, 24–36 h, 48–60 h, and >60 h, and showed that each 10-hour delay increased the odds ratio by about 5%. However, in their study, the one-year mortality rates were 23% and 24% in the 36–48 h and 48–60 h groups, respectively.

It is well known that prolongation of the preoperative period delays the inflammatory response, and increases the rates of pneumonia and decubitus ulcers [19] . Although an increase in the mortality rate is expected in patients admitted on weekends and holidays, several studies show that hospitalization on weekdays or weekends did not affect mortality [6,20] . In our study, mortality increased with an increase in the ASA score, but there were no statistically significant differences between weekday and weekend admission groups concerning mortality rates, which is consistent with the literature.

The impact of postoperative care models on mortality and morbidity is an essential topic. Several care models, extending from orthogeriatric to conventional models, are implemented in geriatric fracture centers and geriatric care units. Patients with hip fracture who are hospitalized in orthopedic clinics are followed up with traditional follow-up models [21] . They are monitored in the clinic until surgery and referred to the ICU if the anesthesiologist considers it necessary during the operation (this depends on personal experience and lacks objective criteria). As referrals to the ICU are not based on objective criteria, in studies conducted with conventional follow-up models, the impact of follow up on mortality rate is hard to standardize and assess.

Although the effects of orthogeriatric models on the treatment of complications and decreased hospitalization durations [22] have already been demonstrated, it is not yet clear whether these approaches have reduced mortality or not [13,22,23]. Furthermore, most of the centers do not have geriatrists or geriatric units. Duaso et al. [13] compared the conventional model with the acute geriatric unit (ACU) model in hip fractures and found that there was no statistically significant difference for the 6-month and 12-month mortality rates, even though the length of stay (LOS 15.76 days vs. 5.9 days; P < 0.001) and hospital mortality (4.5% vs. 1.3%; P < 0.01) were lower in the ACU group compared to the conventional group. In our study, all patients were referred to the ICU in the postoperative period. Later, they were followed up in the orthopedic clinic.

In contrast, Beaupre et al. [9] recently published a study that suggested increased 30-day and 3-month mortalities with the prolongation of time until surgery in patients with hip fractures. However, they stated that delays of less than 40 h and age older than 85 years showed a profound impact on increased mortality.

As far as we know, mortality is affected by the type of fracture and by the surgical method implemented in proximal femur fractures [6,24]. For example, fractures of the femur neck have lower mortality rates compared to trochanteric fractures [6]. However, most studies evaluate mortality without distinguishing between the fracture types or the surgical method implemented [8,9]. In most of the published studies, the duration between admission and surgery was measured. Mortality was evaluated in relation to those measurements. However, mortality may change as a result of transport time to the hospital, the population, and the geographic area for which the hospital is responsible. Time spent in a small health center before transfer to major health hospitals is probably not taken into consideration in these studies, which may, in fact, have some impact on mortality rates.

The implementation of the same surgical and anesthetic methods for the same fracture types, and the same postoperative follow-up model for all patients, some are strengths of our study. However, the relatively small sample size and the retrospective study design, using only one center, may be considered as limitations of the study. Also, the study did not involve a comparison of surgical methods nor a financial analysis.

## 5. Conclusion

In summary, a short period between admission and surgery is ideal in geriatric hip fractures. However, putting a cut-off point of less than 48 h would be rather empirical. The surgery must be planned appropriately according to the capacity of the hospital, the number of operation rooms, and the workload of the surgical team. It seems that the mortality rate is not affected by time in a model in which all patients are transferred to the hospital within the first day and are operated in less than 96 h. Regarding the timing of early surgery in geriatric hip fractures, new studies focusing on the effects of fracture types, treatment methods, and postoperative care on mortality are needed. Although shortening the time until the surgery seems to be crucial in decreasing mortality following hip fractures, we did not find any difference considering a cut-off of 48 h.
